# Supplementary material for: Patterns of Intron Gain and Loss in Fungi
Source: PLoS Biol. 2004 Nov 30;2(12):e422. doi: 10.1371/journal.pbio.0020422 (PMC532390; doi:10.1371/journal.pbio.0020422)
Supplement: Table S1 — Also available at http://genes.mit.edu/NielsenEtAl/. (4.3 MB ZIP). [file pbio.0020422.st001.zip › NielsenEtAl/html/853.html]

AN3169.1.NCU01786.1.MG06678.1.FG10840.1


```
 CLUSTAL W (1.82) Multiple Sequence Alignments - Introns Inserted


Sequence 1: NCU01786.1	431 aa
Sequence 2: MG06678.1	438 aa
Sequence 3: FG10840.1	456 aa
Sequence 4: AN3169.1	435 aa
Alignment Length: 476 aa
Number Identitical Residues: 214 aa
Alignment Score (without introns) 10295


MG06678.1 	MVRNIVVLGGNSHPELVDEVCGILSIPPCDRILSKFEVGESRCEIKDSVRGKDVYIIQSG
NCU01786.1	MVRNIVVLGGNSHPELVESICGILGLPACSRILTKFSSGESRCEIQDSVRGKDVYIIQTG
FG10840.1 	MVRNIVVLGGNSHPQLTENVCHILGVPASNRILGKFSGGESRCEIKDSVRGKDVYIIQSG
AN3169.1  	MVRNIVVIGGTSHPQLTQTICDILGIPPADVLLSKFSVGETRVEIKESVRGKDVYILQSG
          	*******:**.***:*.: :* **.:*... :* **. **:* **::*********:*:*

MG06678.1 	----GGKSNDHFIELCIS------G2SAKRVTAVLPLFPYSRQPDLPYKKSGAPLSQAAG
NCU01786.1	FGGNGSRLNDHFMDLCIMISACKTG~SARRVTAVLPLFPYSRQPDLPYNKAGAPLYKAPT
FG10840.1 	----SGNVNDNLIDLCIMISACKTG~SAKRVTAVVPLFPYSRQPDWPYNKAGAPLSQISG
AN3169.1  	----GGKVNDHLLELLITISACKTA~SARRVTAVLPLFPYSRQSDIPYNKSGAPLVKSSL
          	    ... **::::* *  ::..:. **:*****:********.* **:*:**** : . 

MG06678.1 	SS----NKEYTFESVPATPAPGIPRTAGLTNGVD-ITHMLRDSS-FTNGNAPASPRPAPP
NCU01786.1	PNGENGKNQYTFDSVPPTPGPGVPKTSGLTNSSD-IANQMIKNA-LTNG-----------
FG10840.1 	AS-----KDYTFESVPPTPRPGGPKSAGLPNGVNNLTEKLSKTAEVANGVNGTNGHPTPR
AN3169.1  	EG-KGASNGYTFESTPTTPHPGKLEVGNPINGVS--IDNLQKSLAKAQLEESSGSPVKKR
          	 . ....: ***:*.*.** **  . ..  *. .   . : ..   ::    :.      

MG06678.1 	-SEQFSGKNDANGVNGSRSGKPKAHNSSASTSSLVRSYTTHDYENPSFINHFQARPGYKQ
NCU01786.1	-----SSKE---------------------------QYTTHDYENLSIVNGFQAKPGYKQ
FG10840.1 	RSDTISSTTSEN----------RGHHPENSTSSQRNAYTTHDYENQSNISVFQPKPGYKQ
AN3169.1  	LPNGLSRSDTMES--------------LKSDSSKSTLPNGLLDDSQDKINKFEPRPGYKQ
          	 ..  * .   ..                : ::     .    :. . :. *:.:*****

MG06678.1 	WVAQAGTLVANLLTCAGADGI~ITLELHDHQY~QGFFDIPVDNLYGKPLLQRYIQTHVPN
NCU01786.1	WVAQAGTLVANLLTCAGCDHV1ITMDLHDPQV1QGFFDIPVDNLYGQPLLKRYIQQHIPN
FG10840.1 	WIAQAGTLVADLLTCAGADHI~ITMDLHDPQY~QGFFDIPVDNLYGKALLQNYIQTNIQG
AN3169.1  	WVAQAGTLVADLLTCAGADHV~ITMDLHDPQY~QGFFDIPVDNLYGRPLLKNYIMRNIAN
          	*:********:******.* : **::*** *  *************:.**:.**  :: .

MG06678.1 	YKDAIIVSPDAGGAKRATAIADSLGMDFALIHK~-------------------ERRPTKI
NCU01786.1	WRDAVIISPDAGGAKRATAIADSLGMEFALIHK~VNALLQISPPGLPLNQESQERRPTKI
FG10840.1 	HQNAVIVSPDAGGAKRATLIADSLKTDFALIHK~VIPTTDLSHDDQSSFNASKERRPIRF
AN3169.1  	YKEAVVVSPDAGGAKRATAIADSMGMEFALIHK0-------------------ERRPTRI
          	 ::*:::*********** ****:  :******                    **** ::

MG06678.1 	SDNQNATMMLVGNVSGRICILVDDLCDTGNTITRAAKLLKKQGATYIYALVTHGLFSGDA
NCU01786.1	TDRQNASMMLVGNVTDRVCILLDDIADTGNTITRAAKLLKKEGATTIYALLTHGVFSGDA
FG10840.1 	TEHRNASMMLVGDVSNRICILVDDIIDTGNTITRAAKLLKKEGATKVYALVTHGVFSGDS
AN3169.1  	TDRQNATMMLVGDVKDRTAILIDDLADTSNTITRAAKLLKKEGASQVYALVTHGILSGDA
          	::.:**:*****:*..* .**:**: **.************:**: :***:***::***:

MG06678.1 	IPRINASSIDKIVTTNSVPQAEHK-ALCPKMEILSVSPVFAE~AIRRVHHGESISVLFQH
NCU01786.1	ISRVKASAIDKLVVTNSVPQDEHKKQLGSKLDVLDISPIFAE~AMRRVHHGESISVLFNY
FG10840.1 	IARINASSIDKMLVTNSVPQEEHR-RLCPKLEVLDISPVFAE~AIRRVHHGESISVLFQH
AN3169.1  	IDRINASALDKVVVTNSVSQTEHL-RRCPKLEVLEVGHVFAE0AIRRVHHGESISALFQY
          	* *::**::**::.****.* **     .*:::*.:. :*** *:**********.**::

MG06678.1 	-
NCU01786.1	E
FG10840.1 	N
AN3169.1  	E
          	.
```
